# Spectacles

**Published:** 1858-11

**Authors:** 


### SPECTACLES.

As people get old, the eye flattens, and it becomes necessary, in order to see clearly, and without an effort, to put the thing looked at farther from the eye, or to employ means to make up for the flattening of the eye; hence, the use of spectacles. To look at them, they appear to be nothing more than a flat piece of glass; but, on examination, they are convex, that is, bulge out a little; and the skill in adapting glasses to the eye, consists in selecting those that have a convexity which will just make up for the flattening of the eye, and thus place things very nearly in their natural condition, so that the wearer may see easily without a strain. To do this systematically, glasses are numbered rationally by the French, arbitrarily and bunglingly by the English and Americans. The first glasses used are called No. ninety-six, that is, at ninety-six inches, or eight feet, the rays of light coming through such a glass, meet in a focus—and thus cause distinct vision. The next number is eighty-four, or seven feet, and this increase is so slight, that some opticians never make that number; but fall down to eighty, then seventy-two, after which the numbers decrease slower, as sixty-six, fifty-four, and so on. But, not to perplex the learner of spectacles with any numbers, it is sufficient, for general practical purposes, to be governed by this rule—whenever you begin to feel yourself reading with an effort of the eye, do not buy at once a pair of spectacles, but cease reading or sewing for a moment or two, and look at some distant object; this rests the eye, and enables it to return to the page with great ease; and the instant there is a consciousness of effort, repeat the same process. Doing this judiciously, the use of spectacles may be deferred for a year or more. Then, when weariness comes at once, or when you find yourself instinctively going towards a better light to read your favorite paper, or increasing its distance from the eye, or turning it slightly, so as to get more light on the page, it is high time for glasses—when you should begin with the highest number possible, so that it enables you to read with ease. Never tell the optician your age. It is none of his business. There is as much difference in the eyes as in the faces of men—some are ten years younger than in reality. A man of fifty may require number twenty-four, while his neighbor, of the same age, can see just as well with thirty-six. When you have taken your first number, use it as long as you can, without getting a lower one. But as soon as you perceive an evident strain, a decided effort to see clearly, then get a lower number at once, such a number as will enable you to see easily, without the consciousness of straining.

When beginning to use glasses, use them as short a time as possible, only in deficient light, or on minute objects, and then change the strain to distant or larger objects. By a judicious attention to these two points, the age of the sight will be retarded many years. And, as reading is one of the luxuries of age, one of its most delightful pastimes and amusements, we cannot be too careful of the eye-sight, and should study how we may best husband its powers.

Reader, we speak theoretically, not practically. We don't wear specs yet, nor do we know when we will, for we have nursed our naturally weak eyes for a quarter of a century, and are fully paid for all our care, for numbers of our school-mates of equal age have been using glasses for many years, ten, at least.

